# P02.174. Effectiveness of acupuncture in improving perioperative patient centered outcomes: a comparative effectiveness study

**DOI:** 10.1186/1472-6882-12-S1-P230

**Published:** 2012-06-12

**Authors:** S Attias, E Schiff

**Affiliations:** 1 Bnai Zion Medical Center, Haifa, Israel

## Purpose

Perioperative symptoms such as pain and anxiety are common in spite of standard of care. Such symptoms are associated with a negative surgery experience, and moreover, are correlated with increased perioperative morbidity. The aims of this study were to evaluate whether acupuncture as an add-on to standard of care improves these symptoms. In addition, we assessed whether outcomes are correlated with expectations from CAM.

## Methods

We conducted a pragmatic trial of 479 adult patients undergoing various abdominal operations. Eighty-nine patients received standard medical care, and 390 patients received acupuncture on top of standard medical care, according to patient preference and practitioner availability. Numeric VAS scores for anxiety, pain, nausea and well-being were collected pre and post treatment. Acupuncture was provided according to Traditional Chinese Medicine syndrome categorization.

## Results

There was a significant reduction of VAS scores for all outcomes in the acupuncture group: Anxiety scores were reduced from 4.4 to 2.6 (n=394, p<0.0001), pain from 4.8 to 2.5 (n=380, p<0.0001), nausea from 2.7 to 1.4 (n=380, p<0.0001) and well-being improved from 5.1 to 6.8 (n=384, p<0.0001). Symptomatic improvement was significantly better in the acupuncture group as compared to the standard of care group for all parameters p<0.0001. In the subgroup of patients experiencing moderate to severe symptoms, improvement was even more prominent: anxiety scores were reduced from 6.9 to 3.8 (n=214, p<0.0001); pain from 6.9 to 3.6 (n=231, p<0.0001); nausea from 7.2 to 3.3 (n=111, p<0.0001); and well-being improved from 3.7 to 6.2 (n=252, p<0.0001). We did not find a correlation between outcomes and patients’ expectations regarding acupuncture.

## Conclusion

Our results demonstrate that acupuncture therapy significantly improved common symptoms in patients undergoing surgical interventions.

